# Changing Patterns of Undiagnosed HIV Infection in the Netherlands: Who Benefits Most from Intensified HIV Test and Treat Policies?

**DOI:** 10.1371/journal.pone.0133232

**Published:** 2015-07-17

**Authors:** Eline L. M. Op de Coul, Imke Schreuder, Stefano Conti, Ard van Sighem, Maria Xiridou, Maaike G. Van Veen, Janneke C. M. Heijne

**Affiliations:** 1 Centre for Infectious Diseases Control, National Institute for Public Health and the Environment, Bilthoven, The Netherlands; 2 Department of ViroScience, Erasmus Medical Centre, Rotterdam, the Netherlands; 3 Statistics, Modelling and Economics Department, Public Health England, London, United Kingdom; 4 Stichting HIV Monitoring, Amsterdam, The Netherlands; 5 Cluster Infectious Diseases, STI clinic department, Amsterdam Health Service, Amsterdam, the Netherlands; David Geffen School of Medicine at UCLA, UNITED STATES

## Abstract

**Objectives:**

To estimate HIV prevalence, the number of people living with HIV/AIDS (PLWHA) and the undiagnosed proportion in the Netherlands for 2012, and to compare these with published 2007 estimates.

**Design:**

Synthesis of all available data sources.

**Methods:**

Multi-Parameter Evidence Synthesis (MPES) was used to obtain estimates in mutually exclusive key populations at higher risk in three geographical regions (Amsterdam, Rotterdam, rest of the Netherlands). Data sources included HIV prevalence surveys, diagnoses at STI clinics, and registered cases in HIV care. Group specific estimates were reported as Bayesian posterior medians and 95% credible intervals (CrI).

**Results:**

The 2012 model estimated 24,350 PLWHA (95% CrI 20,420–31,280) aged 15–70 years; 2,906 (+14%) more than in 2007. The estimated population HIV prevalence was 0.20% (95% CrI 0.17–0.26%). The overall proportion of undiagnosed HIV was lower in 2012 (34%, 95% CrI 22–49%) compared to 2007 (40%, 95% CrI 25–55%). After MSM, migrants from sub-Saharan Africa and the Caribbean formed the largest groups of PLWHA, but proportions of undiagnosed HIV remained high in these groups, 48% and 44% respectively. Amsterdam had lowest proportions undiagnosed for most key populations at higher risk, including MSM and migrants.

**Conclusions:**

In 2012, the number of PLWHA was higher compared to 2007, while the proportion of undiagnosed HIV was lower, especially among MSM. Higher HIV testing rates, earlier treatment, and an improved life expectancy may explain these differences. HIV interventions need to be expanded in all key populations at higher risk, with special focus on migrants and key populationsliving outside of Amsterdam.

## Introduction

National estimates of the number of people living with HIV/AIDS (PLWHA) and proportions of undiagnosed HIV are important for informing public health strategies. Since the introduction of combination antiretroviral therapy (cART) in 1996, life expectancy in people treated for HIV has increased substantially [1] and consequently the number of PLWHA has increased. People who are unaware of their HIV infection are estimated to contribute up to 50–90% of new HIV infections [2–6] and they are unable to benefit from HIV treatment, which underlines the importance of obtaining insight in this undiagnosed population. It becomes even more important as ‘Test and Treat’ (TT) is emerging as a global prevention strategy and supported by the World Health Organisation and UNAIDS (http://www.who.int/hiv/pub/2009progressreport/en/index.html)

Despite increased HIV testing rates [7] and the wide availability of cART in the Netherlands, there has been no evidence of a sustained decline in new HIV diagnoses [8]. Moreover, the percentage of people unaware of their HIV positive status, estimated at 40% in 2007 [9], and the proportion of HIV patients with late entry into care (i.e. with CD4 cell count <350/mm3 or an AIDS-defining event regardless of CD4 count, quantified at 43% in 2012) are relatively high in the Netherlands [8] compared to other Western countries [10–12]. Although men who have sex with men (MSM) account for most of the new HIV diagnoses (68% in 2012), heterosexuals–especially migrant populations—are at greatest risk for late entry into care [8].

In the past years, various intervention strategies have been implemented to improving HIV testing rates in the Netherlands. First, opt-out HIV screening (test routinely taken unless a person refuses) among pregnant women was introduced in 2004 and in 2010 followed by opt-out testing for HIV in all sexually transmitted infection (STI) clinics since both with an overall test uptakes of 99% or higher [13, 14, 7]. Second, guidelines for repeated HIV testing (every 6 months) were issued for MSM in 2010 [15]. Third, internet facilities for HIV testing and partner notification (PN) were introduced [16, 17] and PN practices for HIV/STI were improved at several STI clinics since 2010 [18, 19]. Last, earlier HIV treatment has been promoted and Dutch treatment guidelines, which follow the US Department of Health and Human Services (DHHS) guidelines, strongly recommend treatment for all HIV-infected individuals with CD4 counts <500/mm^3^, but treatment should be considered for all individuals regardless of CD4 counts [20]. All these strategies likely resulted in increased HIV testing rates among MSM as percentages of MSM reporting to be ever tested for HIV increased from 60% in 2006 to 78% in 2011 [21, 22]. However, it is unclear whether these strategies had any impact on HIV prevalence or proportions of undiagnosed HIV.

The last comprehensive estimate of the number of PLWHA and the proportion of undiagnosed HIV in the Netherlands was conducted in 2007 [9]. Then, various methods were compared, including Multi-Parameter Evidence Synthesis (MPES) methodology [23]. The objective of this study was to update estimates of HIV prevalence, PLWHA and the proportion of undiagnosed HIV for 2012 using MPES methodology. We compared these new estimates with those of 2007 to gain insight in trends in HIV prevalence and proportions of undiagnosed HIV by key population in the past 5 years. Such insights are fundamental for prioritising (new and existing) HIV prevention strategies in certain key populations at higher risk for allocating resources and for a greater offer and/or uptake of HIV testing in specific groups.

## Methods

The MPES methodology combines multiple data sources to produce estimates of subgroup-specific population sizes, HIV prevalence and the percentage of (un)diagnosed persons. Details on the MPES methodology for the Dutch situation are described elsewhere [24, 9] and in the S1D Text. In brief, the target population was restricted to men and women aged 15–70 years living in the Netherlands at the end of 2012. Similar to the 2007 estimates [24, 9], individuals were divided according to place of residence in three non-overlapping regions (Amsterdam, Rotterdam, and rest of the Netherlands) since the Dutch HIV epidemic is geographically clustered. Migrant populations in Amsterdam and Rotterdam are fairly different, compared to each other as well as the rest of the Netherlands, in terms of countries of origin and how these populations are distributed (very centralized in a few areas in Amsterdam versus very scattered throughout the city in Rotterdam). Also, MSM populations are concentrated in these cities compared to the rest of the Netherlands. The three regions accounted for respectively 42, 13 and 45% of the HIV cases registered in care in 2012.

In each region, individuals were subdivided in mutually exclusive key populations ranked by decreasing risk of infection. The group with highest infection risk were MSM, further divided into those attending an STI clinic in 2012 and those not attending an STI clinic, as HIV prevalence and sexual activity levels are expected to differ between these two groups. The third and fourth groups included injecting drug users (IDU) and female sex workers (FSW). Heterosexual STI clinic attendees formed the next group, in turn divided into migrants from Sub-Saharan Africa (SSA), the Caribbean (CRB) (including the former Netherlands Antilles and Surinam, two former Dutch colonies), and the remaining STI clinic attendees. All SSA and CRB migrants not attending STI clinics formed the next two groups. Last, we included individuals who were not in any of the above groups, referred to as individuals at low-risk of infection. Except for MSM and FSW, all groups were stratified by gender. Individuals with multiple risk profiles were categorised in the highest-ranking profile.

### Data

The MPES method requires data input on population size, HIV prevalence and the proportion diagnosed, or a combination of those depending on the data availability for every risk group, gender and region. The proportion diagnosed is based on the number of HIV cases registered in care from the ATHENA observational HIV cohort (the national HIV registry of HIV treatment centres) [8]. A multiplication factor was incorporated to account MSM who were diagnosed but did not enter HIV care yet based on an internet survey performed among MSM [22].

S1B Text provides an overview of all data (sources) used, and indicates which data (sources) are updated compared to the 2007 estimate. Where possible, the same data sources were used as for the 2007 estimate, but with values updated for 2012 (as for STI clinic data). For data sources that could not be updated, comparable alternative data sources were sought (as for data on FSW). If there were no recent data (not even from an alternative source), then we used the same data as in 2007; for example, in 2007 data were used from anonymous surveys among migrants, but there has been no other survey among migrants in the Netherlands. Therefore, the same data were used in both estimates. For migrants visiting STI clinics, data could be updated, but a new definition for ethnicity was applied to the national registry of STI clinics in 2010/2011 [25]. The definition changed from self-reported ethnic group to ethnicity based on the country of birth of the attendee and his/her parents, consistent with the definition from Statistics Netherlands [26]. Therefore, STI clinic attendees from SSA and CRB, who identify themselves as Dutch, were classified as Dutch in the 2007 estimate and as migrants in the 2012 estimate.

### Changes in the MPES model

For some key populations or data sources new insights became available that led to five changes in the model. First, in the 2007 estimate, all individuals who ever injected drugs were included in the group IDU. However, many individuals who injected drugs in the past may have stopped injecting as evidenced by the tremendous decline in the number of current injectors, the decrease in transmission of HBV, HCV, and HIV in this group, and the unpopularity of injecting drugs since the 90’s in the Netherlands [27–29]. Therefore, we used a new definition of IDU in 2012, including only those who reported injecting drugs in the last six months (current IDU). In contrast to the 2007 model, for Amsterdam and Rotterdam, HIV prevalence data for IDU were not available in 2012. Therefore HIV prevalence data for problematic drug users were used that include IDU and people using opioids, cocaine and/or amphetamines on a regular basis. Similar to the 2007 model, all other data sources involved IDU only. Second, to better reflect the number of cases in HIV care we excluded cases that were lost to follow-up since they might have died or moved to other countries. Third, since blood donors form a specific group with extremely low HIV positivity (i.e. there were no HIV diagnoses in 2012 among blood donors) [30], they were no longer utilised to estimate HIV prevalence in low-risk populations. This is consistent with recent developments in estimating HIV prevalence using MPES in the UK [31]. Fourth, we have changed the opting-out prevalence in heterosexual non-migrant STI clinic attendees to zero in response to a change in HIV testing policy at STI clinics. Attendees aged ≤24 years with no additional risk factors are no longer routinely tested for HIV in STI clinics; only those tested positive for *chlamydia trachomatis* are additionally tested for other STI. In 2012, no HIV infection was diagnosed in that group [32]. Since the majority of heterosexuals opting out of HIV testing were young non-migrant STI clinic attendees, we assumed that the heterosexual non-migrant opting-out group as a whole is characterised by lower risk of HIV infection. Last, new data were available in 2012 for pregnant women in Rotterdam and the rest of the Netherlands that were not available in 2007 (in 2007 only data in Amsterdam was available). By combining data from antenatal screening [33] and the ATHENA cohort [8], we obtained data on the proportions diagnosed for migrant and non-migrant pregnant women. We were also able to make a more accurate distinction between Dutch, SSA and CRB pregnant women in the rest of the Netherlands compared to the Dutch/non-Dutch breakdown (including many other migrant groups) in 2007. The data from non-migrant pregnant women are used to inform HIV prevalence in the low risk population.

### Simulations

As in Conti *et al*. [24], the model was fitted within a Bayesian estimation framework using the freely available WinBUGS MCMC simulation engine [34]. The simulation was started at three independent initial states with convergence determined visually after 30,000 iterations. After the first 30,000 iterations, a 30,000-sized sample from the full posterior distribution was subsequently extracted, with estimates reported as medians and 95% credible intervals (CrI). The code, data and initialisation files required to produce the model estimates are provided as S2–S4 Texts.

### Sensitivity analysis

Although we believe that our changes to the model are a better reflection of the current HIV situation in the Netherlands, for ease of comparison, we performed a sensitivity analysis in which we reversed the five previously described model adaptations back to the 2007 set-up (S1D Text). We compared the overall HIV prevalence, PLWHA and the proportion undiagnosed with the current estimates. We then changed every model assumption one by one to examine which assumption was most sensitive to changes in the model output.

### Ethics statement

This study was based on secondary analysis using published data and data from the ATHENA national observational HIV cohort. The cohort includes anonymized data from all HIV-infected patients living in the Netherlands who receive care in one of the 27 HIV treatment centres. Written informed consent and ethical approval is not required, as data collection is part of HIV care.

## Results

### HIV prevalence and number of PLWHA

An estimated 24,350 (95% CrI 20,420–31,280) people aged 15 to 70 years old were living with HIV in the Netherlands as of December 2012 (S1C Text). This number represented an overall HIV prevalence of 0.20% (95% CrI 0.17–0.26%): 0.33% in males (95% CrI 0.27–0.45%) and 0.07% in females (95% CrI 0.07–0.08%). There were 2,906 more PLWHA (+14%) in 2012 compared to 2007.

**Fig 1:**
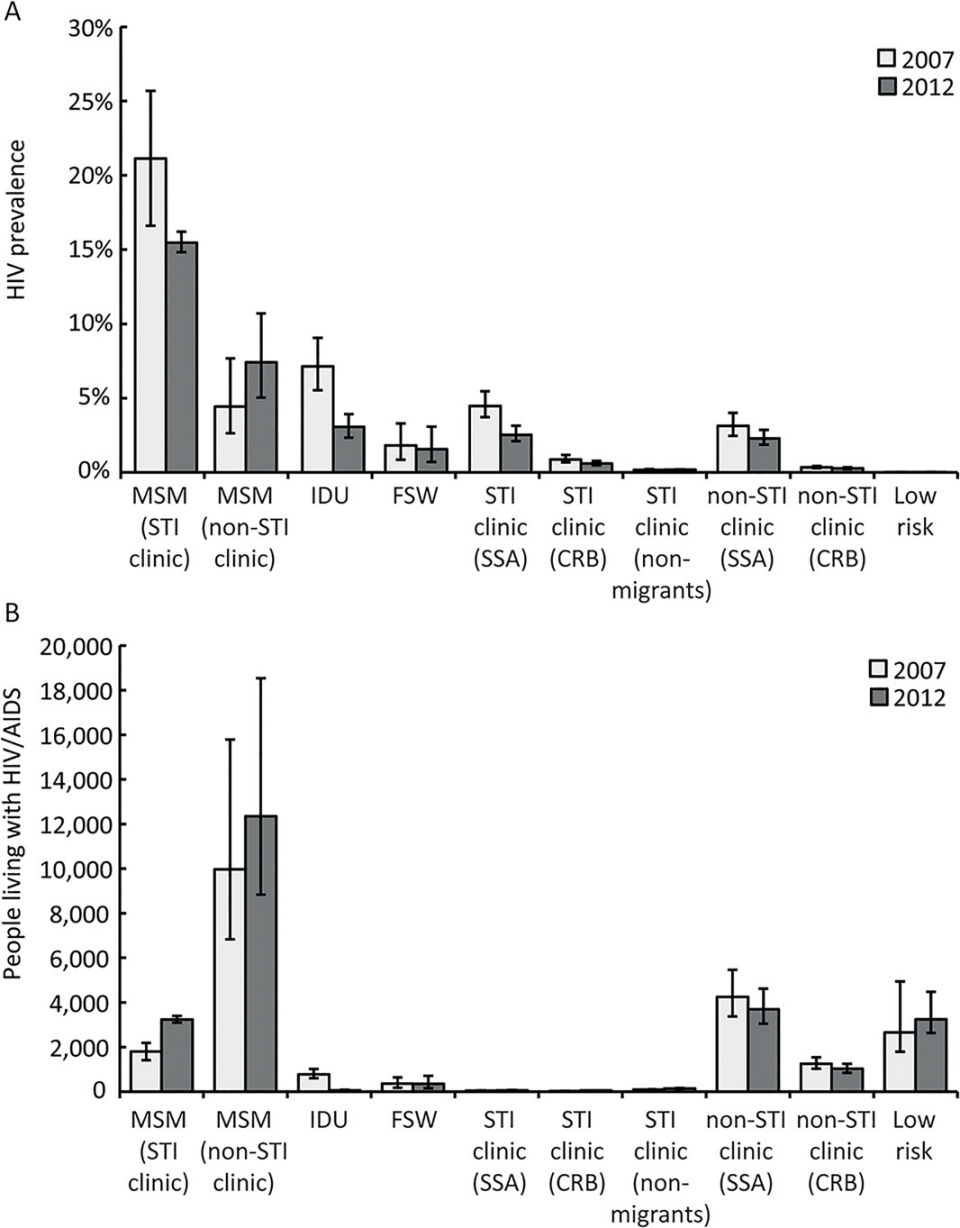
Estimates of HIV prevalence (A) and people living with HIV/AIDS (B) by key population at higher riskin the Netherlands for 2007 and 2012. Vertical lines within bars represent 95% credible intervals. MSM: men having sex with men; IDU: injecting drug users; FSW: female sex workers; SSA: Sub-Saharan Africans; CRB: Caribbeans.

An estimated 8.3% (95% CrI 6.1–11.3%) of all MSM were HIV positive in 2012, corresponding to approximately 15,590 (95% CrI 12,070–21,780) MSM living with HIV in the Netherlands (33% higher compared to 11,758 in 2007). HIV prevalence was much lower in 2012 among MSM attending STI clinics (15.5% vs. 21.1% in 2007), although the number of PLWHA in MSM attending STI clinics was higher in 2012 (3,237) compared to 2007 (1,800) (Fig 1A and 1B), reflecting the increase in testing rates among MSM. The number of HIV infected MSM not attending STI clinics also became larger, from an estimated 9,965 in 2007 to 12,350 in 2012 (24% higher).

The second largest group of PLWHA in the Netherlands were migrants from countries with a generalised HIV epidemic (4,844; 95% CrI 4,006–6,009 in 2012). An estimated 2.3% of SSA migrants in the Netherlands were HIV infected (3,761 persons) and 0.3% of migrants of CRB origin (1,083 persons). HIV prevalence among SSA not attending STI clinics (2.3%) was similar to that estimated among SSA attending STI clinics (2.5%). In the latter group, a lower HIV prevalence was observed compared to 2007. In the CRB population, HIV prevalence was estimated at 0.6% for STI clinic attendees and 0.3% for people not attending STI clinics.

Almost 1.6% of FSW were HIV positive in 2012, corresponding to 351 FSW living with HIV in that year. The 2012 HIV prevalence rates in non-migrant STI clinic attendees (0.2%) and in the low-risk population (0.03%) did not change notably compared to 2007.

### Undiagnosed HIV infections

**Fig 2:**
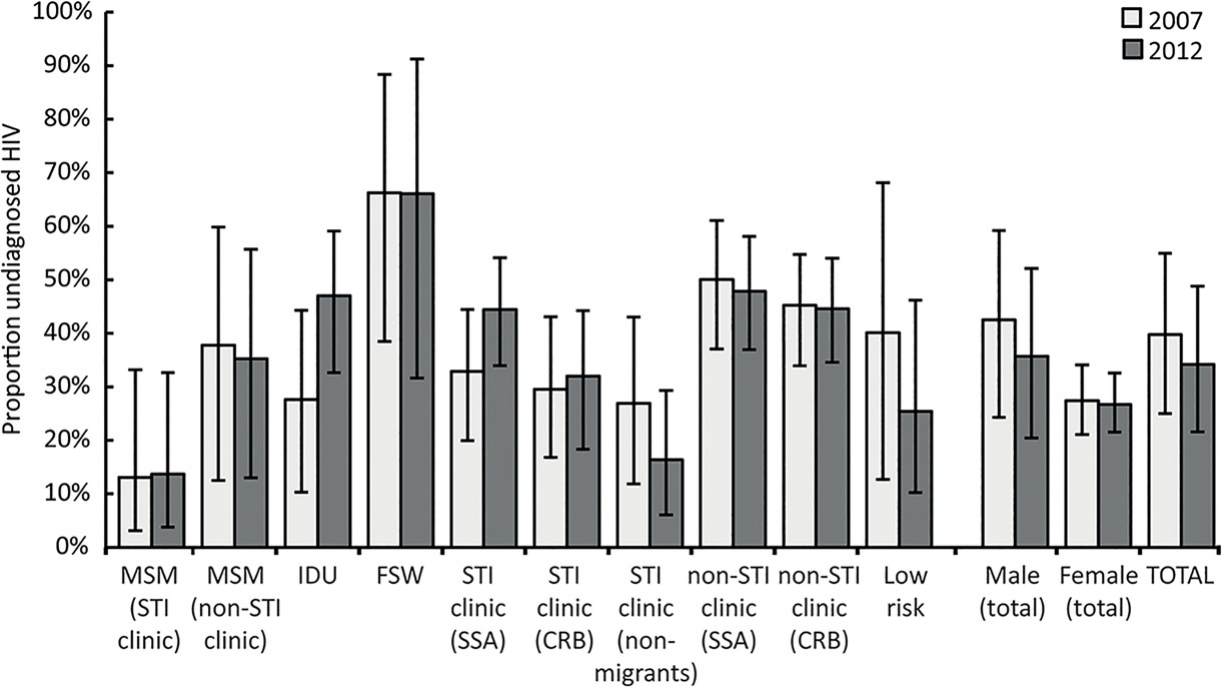
Estimates of the percentage of HIV infected individuals unaware of their HIV infection by key population at higher riskin the Netherlands for 2007 and 2012. Vertical lines within bars represent 95% credible intervals. MSM: men having sex with men; IDU: injecting drug users; FSW: female sex workers; SSA: Sub-Saharan Africans; CRB: Caribbeans.

Overall, an estimated 34.2% (95% CrI 21.6–48.8%) of HIV infected individuals were unaware of their infection in 2012 (Fig 2). This corresponds to 8,318 (95% CrI 4,406–15,260) undiagnosed individuals, a decrease of 202 individuals compared to 2007 (S1C Text). Among men, the proportion undiagnosed was 36% in 2012 while 43% in 2007, but among women no changes were observed (both 27%). Of all key populations at higher risk, percentages of undiagnosed HIV were lowest for MSM attending STI clinics (14%) and non-migrant STI clinic attendees (16%).

High proportions of undiagnosed infections were found among SSA or CRB populations not attending STI clinics (48% and 45% respectively). In some groups, small declines in percentages of undiagnosed infections were seen relative to 2007, as for the overall MSM group (from 34% to 31%) and non-migrant STI clinic attendees (from 27% to 16%). In other key population at higher risk (IDUs, FSW, SSA and CRB not attending STI clinics) the proportion of undiagnosed HIV remained stable but high or could not be compared to 2007 due to definition changes in the 2012 model.

### Regional differences

Notable differences in HIV prevalence and proportions of undiagnosed HIV for the two largest groups of PLWHA (MSM and migrant populations) were found between regions (Table 1). In Amsterdam, the overall HIV prevalence was estimated to be higher (0.9%) than in Rotterdam (0.5%) and the rest of the Netherlands (0.1%). Of MSM living in Amsterdam in 2012, 14% were estimated to be HIV infected, while these proportions were 11% in Rotterdam and 7% in the rest of the Netherlands. Differences in HIV prevalence among migrant populations across regions were less profound.

**Table 1:** Estimates of HIV prevalence and proportions undiagnosed for MSM and migrants by region in the Netherlands for 2007 and 2012.

	Prevalence (%) (95% Crl)		Proportion undiagnosed (%) (95% Crl)		Relative difference proportion undiagnosed (%)
Key population	2007	2012	2007	2012	
**TOTAL**	**0.2 (0.1–0.2)**	**0.2 (0.2–0.3)**	**39.7 (25.0–55.0)**	**34.2 (21.6–48.8)**	**-14.0**
Amsterdam	0.9 (0.8–1.0)	0.9 (0.9–1.0)	17.3 (10.8–26.4)	13.6 (8.3–21.8)	-21.3
Rotterdam	0.6 (0.5–0.7)	0.5 (0.5–0.6)	45.8 (34.6–57.4)	42.8 (34.4–53.0)	-6.6
Rest of NL	0.1 (0.1–0.2)	0.1 (0.1–0.2)	46.6 (24.3–64.9)	39.9 (21.1–57.6)	-14.5
**MSM**	**5.1 (3.2–8.3)**	**8.3 (6.1–11.3)**	**34.3 (11.1–56.0)**	**31.2 (11.3–50.7)**	**-9.2**
Amsterdam	11.3 (9.6–13.2)	13.7 (12.4–15.2)	10.4 (4.4–21.9)	8.6 (3.3–18.3)	-16.7
Rotterdam	9.3 (7.1–11.6)	11.0 (9.6–12.5)	17.9 (3.4–38.5)	31.2 (22.3–40.3)	+74.6
Rest of NL	3.9 (2.0–7.5)	7.0 (4.3–10.6)	46.5 (10.5–69.9)	40.4 (9.2–62.8)	-13.2
**SSA**	**3.1 (2.5–4.0)**	**2.3 (1.9–2.9)**	**49.9 (37.0–60.9)**	**47.8 (36.9–58.1)**	**-4.1**
Amsterdam	2.7 (2.2–3.6)	2.2 (1.8–2.8)	30.9 (18.7–47.6)	32.6 (21.0–45.4)	+5.4
Rotterdam	2.6 (1.9–3.6)	1.9 (1.4–2.5)	63.8 (51.0–74.0)	56.4 (44.0–66.9)	-11.6
Rest of NL	3.4 (2.4–4.7)	2.4 (1.8–3.2)	49.7 (30.4–63.9)	49.0 (34.0–61.5)	-1.4
**CRB**	**0.4 (0.3–0.4)**	**0.3 (0.2–0.4)**	**44.8 (33.7–54.3)**	**43.9 (34.0–53.3)**	**-2.0**
Amsterdam	0.4 (0.3–0.5)	0.4 (0.3–0.5)	22.2 (10.8–40.4)	24.5 (12.4–38.9)	+10.3
Rotterdam	0.5 (0.4–0.8)	0.5 (0.3–0.6)	58.7 (45.0–70.2)	52.0 (38.8–64.0)	-11.4
Rest of NL	0.3 (0.2–0.4)	0.2 (0.2–0.3)	45.6 (25.7–60.3)	46.9 (30.5–60.6)	+3.0

MSM: men having sex with men; SSA: Sub–Saharan Africans; CRB: Caribbeans; CrI: Credible interval; NL: Netherlands.

Amsterdam had the lowest overall proportion of undiagnosed HIV among MSM and migrant populations in 2012 (Table 1). For the overall SSA and CRB populations, the proportion undiagnosed HIV was considerably lower in Rotterdam compared to 2007, but we observed hardly any changes over time in the estimated proportions of undiagnosed HIV among migrants in Amsterdam and the rest of the Netherlands.

### Sensitivity analysis

The estimated HIV prevalence was not sensitive to the model alterations. The number of PLWHA increased from 24,350 to 25,150 (95% CrI 21,670–32,690) based on the 2012 and 2007 model assumptions respectively, and the proportion undiagnosed decreased from 34.2% using the 2012 assumptions to 30.4% (95% CrI 19.3–46.5%) using the 2007 assumptions. However, the model fit to the data degraded when using the 2007 model assumptions. The model outcomes were most sensitive to the definition change of IDU from ever injected to recent injectors (S1D Text).

## Discussion

Using the MPES method, we estimated that 24,350 people aged 15–70 years were living with HIV/AIDS in the Netherlands by the end of December 2012. Compared to 2007, this estimate was 14% higher. The number of MSM living with HIV/AIDS was 15,590 in 2012, 33% higher than in 2007. The overall proportion of undiagnosed HIV among MSM was one of the lowest of all key populations at higher risk, slightly under the overall proportion undiagnosed of 34.2%, but still as high as 31%. Among migrant populations, proportions of undiagnosed HIV were estimated to be excessively high; 44% in the CRB population and 48% in people from SSA.

One of the strengths of MPES is its ability to synthesise all the available data sources including HIV sero-prevalence data, population size estimates and registered HIV cases. Furthermore, MPES allows checking for inconsistencies between different data sources; a tool not offered by other estimation methods [35, 9]. Another strength of this study is the inclusion of region-specific data, providing estimates for separate regions with their own epidemiological characteristics. There are also limitations to the study. First, improvements were made to the 2012 model to better reflect the current epidemiological situation and to improve the model fit to the data, but this might have hampered the comparison with 2007. However, sensitivity analysis showed that our estimates of the undiagnosed fraction are conservative and that the model was most sensitive to changing the definition of IDU from ever injecting to recent injectors. We believe that current injectors are a better reflection of the IDU situation in the Netherlands as the number of IDUs has decreased substantially since [27, 28, 8]. Another limitation is that, for all regions, information on HIV prevalence among migrants not attending STI clinics could not be updated due to lack of prevalence surveys in this group. For the rest of the Netherlands, recent data were also lacking for IDU and FSW. Therefore, observing no change in those groups does not necessarily mean that no change has occurred since 2007. Last, as in 2007, no information was available on undocumented persons living in the Netherlands.

The estimated number of PLWHA is very close to other estimates for the Netherlands. Our estimate of 24,350 PLWHA is consistent with the UNAIDS estimate for the Netherlands of 20,000–34,000 PLWHA in 2012 [1], even though a different method was used (i.e. the Spectrum method). Our estimate of the proportion diagnosed (66%) also coincides with an estimated 68% of diagnosed HIV infected individuals as of June 2013 from the Dutch HIV monitoring foundation [8], which was based on dividing the number of people in care by the UNAIDS estimate of PLWHA in 2011 [36]. However, a distinction between key populations at higher riskand regions could not be made.

We showed that MSM were the largest group of PLWHA, followed by migrants. The smaller proportion of undiagnosed HIV among MSM in 2012 compared to 2007 might be associated with recent HIV prevention interventions in this group. There has been an increasing emphasis on HIV testing: testing rates [32] and testing frequency increased [37] and PN practices improved [19]. The proportion of undiagnosed HIV among migrants continued to be high. Although this can be partly explained by the lack of updated data for migrant groups, also other data showed that migrant populations are less well reached for HIV testing [8, 38]. The smaller proportion of undiagnosed HIV among MSM and the continuing high rates of undiagnosed HIV among migrants are also in line with results from a Dutch study showing that proportions of patients with late entry into care decreased over time in MSM, but not in migrant populations [39], indicating that migrant populations are less well reached for HIV testing as other subpopulations in the Netherlands. We found that proportions of FSW unaware of HIV remained stable at a high level, most probably because FSW are hard-to-reach for HIV testing as they are for HBV vaccination [40]. In that program, FSW are more difficult to reach than MSM because of high migration rates and short-stay in the Netherlands.

We observed large regional differences in proportions of people unaware of their HIV infection between Amsterdam and the other regions. Although this could be explained by sparser or missing data sources in the other regions, the regional differences were quite remarkable. Reasons for these differences could be attributed to a more active HIV testing policy and a longer history of opt-out testing in Amsterdam. In Amsterdam, pregnant women are screened for HIV since 1988, while the national screening program was only implemented in 2004 [33]. Opt-out testing for HIV at the Amsterdam STI clinic was implemented in 2007 [14], while this policy was introduced nationwide in STI clinics in 2010 [7, 13]. Also, key populations and health care providers in Amsterdam might have a higher awareness of HIV testing, possibly related to relatively higher HIV prevalence rates and higher numbers of key populations living in the city. The lower percentages of late presentation among newly diagnosed HIV patients in Amsterdam compared to most other regions in the Netherlands might also indicate this higher awareness [32, 39]. In Rotterdam, the percentage of undiagnosed MSM is much higher than in Amsterdam and is also higher than the Rotterdam estimate for 2007, mainly due to a model artefact in the estimates percentage of undiagnosed MSM STI clinic attendees. In this group, the percentage undiagnosed are determined by the people that opt-out for testing. Since the prevalence of HIV is unknown is this group, this will be inferred by the model under the assumption that the prevalence of HIV is higher among MSM that opt-out for testing compared to those opting-in [9]. In Rotterdam, HIV prevalence decreased substantially between 2007 and 2012 among MSM STI clinic attendees opting-in for HIV testing. This makes the contribution of the HIV prevalence from MSM opting-out to the overall HIV prevalence in MSM STI-clinic attendees relatively larger, leading to high fractions undiagnosed among MSM STI clinic attendees.

This study showed the importance of regular updating estimates of the number of PLWHA and of the proportion of undiagnosed HIV to guide policy makers in allocating resources and planning new prevention activities. To facilitate frequent updating, new data to feed into these methods is a prerequisite. This highlights the need of regular population estimates (e.g. FSW, MSM, migrant populations) and population-based sero-surveys.

The lower proportion of undiagnosed HIV in 2012 is possibly associated with higher HIV testing rates. MSM seem to benefit most from these interventions since proportions of undiagnosed HIV in this group showed the largest decline. Despite these positive developments, the annual number of new HIV diagnoses does still not decline visibly. To curb the epidemic, testing, entry and retention into care, and early access to treatment must be scaled up and continuous awareness on sexual risk behaviour must remain [8]. Besides STI clinics, GPs could have a more active role in the detection of HIV, as recently put forth in the Dutch guidelines on STI testing for GPs [41]. To reduce the number of undiagnosed HIV infections, testing efforts need further expansion among all key populations at higher risk, but additional attention must be paid to migrant populations and key populations living outside of Amsterdam.

## Supporting Information

S1 Text (DOCX)

S2 Text (TXT)

S3 Text (TXT)

S4 Text (TXT)

## References

[1] Joint United Nations Programme on HIV/AIDS (UNAIDS). Global Report: UNAIDS report on the global AIDS epidemic 2013 Geneva, Switzerland: UNAIDS 2013.

[2] Bezemer D, de Wolf F, Boerlijst MC, van Sighem A, Hollingsworth TD, Prins M et al A resurgent HIV-1 epidemic among men who have sex with men in the era of potent antiretroviral therapy. AIDS. 2008;22(9):1071–7. 10.1097/QAD.0b013e3282fd167c 18520351

[3] Brenner BG, Roger M, Routy JP, Moisi D, Ntemgwa M, Matte C et al High rates of forward transmission events after acute/early HIV-1 infection. J Infect Dis. 2007;195(7):951–9. 10.1086/512088. 17330784

[4] Hall HI, Holtgrave DR, Maulsby C. HIV transmission rates from persons living with HIV who are aware and unaware of their infection. AIDS. 2012;26(7):893–6. 10.1097/QAD.0b013e328351f73f 22313960

[5] Marks G, Crepaz N, Janssen RS. Estimating sexual transmission of HIV from persons aware and unaware that they are infected with the virus in the USA. AIDS. 2006;20(10):1447–50. 10.1097/01.aids.0000233579.79714.8d 16791020

[6] van Sighem A, Vidondo B, Glass TR, Bucher HC, Vernazza P, Gebhardt M et al Resurgence of HIV infection among men who have sex with men in Switzerland: mathematical modelling study. PLoS One. 2012;7(9):e44819 10.1371/journal.pone.0044819 23024766 PMC3443082

[7] Van Aar F, Koedijk FD, Van den Broek IV, Op de Coul E, Soetens LC, Woestenberg PJ et al Sexually transmitted infections, including HIV, in the Netherlands in 2013 Bilthoven: National Institute for Public Health and the Environment (RIVM) 2014.

[8] van Sighem A, Gras L, Kesselring A, Smit C, Engelhard E, Stolte I et al Monitoring report 2013 Human immunodeficiency virus (HIV) infection in the Netherlands. Amsterdam: HIV monitoring Foundation 2013.

[9] van Veen MG, Presanis AM, Conti S, Xiridou M, Stengaard AR, Donoghoe MC et al National estimate of HIV prevalence in the Netherlands: comparison and applicability of different estimation tools. AIDS. 2011;25(2):229–37. 10.1097/QAD.0b013e32834171bc 21150562

[10] Centers for Disease Control and Prevention. Monitoring selected national HIV prevention and care objectives by using HIV surveillance data—United States and 6 U.S. dependent areas-2010. HIV Surveillance Supplemental Report. 2012;17(3).

[11] Guy RJ, McDonald AM, Bartlett MJ, Murray JC, Giele CM, Davey TM et al Characteristics of HIV diagnoses in Australia, 1993–2006. Sexual Health. 2008;5(2):91–6. 18588771 10.1071/sh07070

[12] Mocroft A, Lundgren JD, Sabin ML, Monforte A, Brockmeyer N, Casabona J et al Risk factors and outcomes for late presentation for HIV-positive persons in Europe: results from the Collaboration of Observational HIV Epidemiological Research Europe Study (COHERE). PLoS Med. 2013;10(9):e1001510 10.1371/journal.pmed.1001510 24137103 PMC3796947

[13] Dukers-Muijrers NH, Niekamp AM, Vergoossen MM, Hoebe CJ. Effectiveness of an opting-out strategy for HIV testing: evaluation of 4 years of standard HIV testing in a STI clinic. Sex Transm Infect. 2009;85(3):226–30. 10.1136/sti.2008.033191 19103641

[14] Heijman RL, Stolte IG, Thiesbrummel HF, van Leent E, Coutinho RA, Fennema JS et al Opting out increases HIV testing in a large sexually transmitted infections outpatient clinic. Sex Transm Infect. 2009;85(4):249–55. 10.1136/sti.2008.033258 19103642

[15] SOA AIDS Nederland. "Strategie voor de aanpak van soa’s en hiv onder MSM in Nederland 2013–2018" [in Dutch]. Amsterdam, the Netherlands: SOA AIDS Nederland 2013.

[16] Götz HM, van Rooijen MS, Vriens P, Op de Coul E, Hamers M, Heijman T et al Initial evaluation of use of an online partner notification tool for STI, called 'suggest a test': a cross sectional pilot study. Sex Transm Infect. 2014;90(3):195–200. 10.1136/sextrans-2013-051254 24391062

[17] Zuilhof W, Koekenbier R, van Empelen P, Vriens P. "MAN tot MAN begint goed" [in Dutch]. In: SekSoa magazine 2009. Available: http://www.soaaids.nl/nl/node/1459.

[18] van Aar F, Schreuder I, van Weert Y, Spijker R, Götz H, Op de Coul E et al Current practices of partner notification among MSM with HIV, gonorrhoea and syphilis in the Netherlands: an urgent need for improvement. BMC Infect Dis. 2012;12:114 10.1186/1471-2334-12-114 22583517 PMC3472393

[19] van Aar F, van Weert Y, Spijker R, Götz H, Op de Coul E, for the Partner Notification Group. Partner notification among men who have sex with men and heterosexuals with STI/HIV: different outcomes and challenges. Int J STD AIDS. 2015;26(8):565–73. 10.1177/0956462414547398 25141854

[20] Dutch Association of HIV-treating physicians. When to start treatment [in Dutch]. Available: http://www.nvhb.nl/richtlijnhiv/index.php/Hoofdpagina.

[21] Hospers HJ, Dorfler TT, Zuilhof W. Schorer Monitor 2006 [in Dutch]. Amsterdam, the Netherlands: Schorer Foundation 2006.

[22] van Empelen P, van Berkel M, Roos E, Zuilhof W. Schorer Monitor 2011 [in Dutch]. Amsterdam, the Netherlands: Schorer Foundation 2011.

[23] Ades AE, Sutton AJ. Multiparameter evidence synthesis in epidemiology and medical decision-making: current approaches. J R Statist Soc Ser A. 2006;169:5–35.

[24] Conti S, Presanis AM, van Veen MG, Xiridou M, Donoghoe MC, Stengaard AR et al Modeling of the HIV infection epidemic in the Netherlands: A multi-parameter evidence synthesis approach. Ann Appl Stat. 2011;5(4):2359–84.

[25] Haasnoot A, Koedijk FD, Op De Coul EL, Gotz HM, van der Sande MA, Van Den Broek IV et al Comparing two definitions of ethnicity for identifying young persons at risk for chlamydia. Epidemiol Infect. 2012;140(5):951–8. 10.1017/S0950268811001336 21767454

[26] Statistics Netherlands. Population and population dynamics. The Hague, the Netherlands. Available: http://www.cbs.nl/en-GB/menu/home/default.htm.

[27] European Monitoring Centre for Drugs and Drug Addiction (EMCDDA). European Drug report: Trends and developments Lisbon, Portugal: European Monitoring Centre for Drugs and Drug Addiction 2013.

[28] van den Berg CH, Smit C, Bakker M, Geskus RB, Berkhout B, Jurriaans S et al Major decline of hepatitis C virus incidence rate over two decades in a cohort of drug users. Eur J Epidemiol. 2007;22(3):183–93. 10.1007/s10654-006-9089-7 17334821 PMC2781102

[29] de Vos AS, van der Helm JJ, Matser A, Prins M, Kretzschmar ME. Decline in incidence of HIV and hepatitis C virus infection among injecting drug users in Amsterdam; evidence for harm reduction? Addiction. 2013;108(6):1070–81. 10.1111/add.12125 23347124

[30] Sanquin. Annual report 2012. Sanquin blood supply, Amsterdam, the Netherlands.

[31] Presanis AM, Gill ON, Chadborn TR, Hill C, Hope V, Logan L et al Insights into the rise in HIV infections, 2001 to 2008: a Bayesian synthesis of prevalence evidence. AIDS. 2010;24(18):2849–58. 10.1097/QAD.0b013e32834021ed 20962617

[32] Soetens LC, Koedijk FD, Van den Broek IVF, Vriend HJ, Op de Coul ELM, van Aar F et al Sexually transmitted infections, including HIV, in the Netherlands in 2012 Bilthoven, the Netherlands: National Institute for Public Health and the Environment (RIVM) 2013.

[33] Op de Coul ELM, Hahné S, van Weert YWM, Oomen P, Smit C, van der Ploeg KPB et al Antenatal screening for HIV, hepatitis B and syphilis in the Netherlands is effective. BMC Infect Dis. 2011;11:185 10.1186/1471-2334-11-185 21718466 PMC3160399

[34] Lunn DJ, Thomas A, Best N, Spiegelhalter D. WinBUGS – A Bayesian modelling framework: Concepts, structure, and extensibility. Stat Comput. 2000;10:325–37.

[35] Walker N, Stover J, Stanecki K, Zaniewski AE, Grassly NC, Garcia-Calleja JM et al The workbook approach to making estimates and projecting future scenarios of HIV/AIDS in countries with low level and concentrated epidemics. Sex Transm Infect. 2004;80 Suppl 1:i10–3. 10.1136/sti.2004.010207 15249693 PMC1765837

[36] Joint United Nations Programme on HIV/AIDS (UNAIDS). Global Report: UNAIDS report on the global AIDS epidemic 2012. Geneva, Switzerland: UNAIDS 2012.

[37] van Aar F, Götz H, op de Coul ELM, van Benthem BHB. Repeated HIV testing among men who have sex with men (MSM) attending STI clinics in the Netherlands [abstract]. Presented at the 8th Netherlands Conference on HIV Pathogenesis, Epidemiology, Prevention and Treatment; November 18, 2014; Amsterdam, the Netherlands 2014.

[38] Lange JM. Editorial commentary: Under the spell of the red queen. Clin Infect Dis. 2013;57(7):1048–50. 10.1093/cid/cit425 23921883

[39] Op de Coul ELM, van Sighem A, Brinkman K, van Benthem BHB, van der Ende M, Geerlings S et al. Factors associated with late presentation and advanced disease of HIV in the Netherlands, 1996–2014 [abstract]. Presented at the 8th Netherlands Conference on HIV Pathogenesis, Epidemiology, Prevention and Treatment; November 18, 2014; Amsterdam, the Netherlands 2014.

[40] van Beek P, Urbanus A, Smits V, van den Kerkhof H, Timen A. "Programma bericht EXTRA: Vaccinatieprogramma hepatitis B-risicogroepen" [In Dutch]. Bilthoven, the Netherlands: National Institute for Public Health and the Environment (RIVM) 2013.

[41] Heijnen A, Hermanussen R, SeksHAG expertgroup on STI HIV and sexuality. "Hiv verdieping naast de NHG Standaard ‘Het Soa Consult’ 2013" [in Dutch]. Available: https://www.nhg.org/standaarden/samenvatting/het-soa-consult.

