# Supplementary material for: Changing Patterns of Undiagnosed HIV Infection in the Netherlands: Who Benefits Most from Intensified HIV Test and Treat Policies?
Source: PLoS One. 2015 Jul 17;10(7):e0133232. doi: 10.1371/journal.pone.0133232 (PMC4505862; doi:10.1371/journal.pone.0133232)
Supplement: S1 Text — (DOCX) [file pone.0133232.s001.docx]

**Changing Patterns of Undiagnosed HIV Infection in the Netherlands: Who Benefits Most From Intensified HIV Test and Treat Policies?**

Eline L. M. Op de Coul , Imke Schreuder, Stefano Conti, Ard Van Sighem, Maria Xiridou, Maaike G. Van Veen, and Janneke C.M. Heijne.

**Supporting Information 1A – D**

**A: MPES methodology**

**B: Data sources**

**C: PLWHA and number undiagnosed**

**D: Sensitivity analysis**

**Supporting Information 1A: MPES methodology**

The MPES methodology combines multiple data sources to produce estimates of subgroup-specific population sizes, HIV prevalence and the percentage of (un)diagnosed persons. Details on the MPES methodology for the Dutch situation are described in [1]. In brief, the population was divided into three non-overlapping geographical regions, r, and into key subpopulation at higher risk, g, ranked by decreasing risk of infection (group 1 was the group at highest risk for HIV infection and the last group with the lowest risk). The groups were "hierarchically exclusive" in the sense that individuals with multiple risk profiles were categorised in the highest-ranking profile, ensuring non-overlap between key populations. For each subgroup and region combination, three basic parameters are to be estimated: $\rho_{gr}$, the proportion of the population in subgroup g in region r; $\pi_{gr}$, the corresponding HIV prevalence; and $\delta_{gr}$, the corresponding proportion of infections that are diagnosed. Direct information on the parameters $\rho_{gr}$, $\pi_{gr}$, $\delta_{gr}$ is sometimes available, but mostly indirect evidence is available in the form of functions of these basic parameters. With MPES, direct and indirect evidence is combined with initial beliefs or expert opinion on the basic parameters, expressed in terms of probability distributions (priors), to produce a ‘posterior’ distribution for the basic parameters and any functions of these. This posterior distribution represents our final knowledge on the quantities of interest. Specifically, let $\boldsymbol{y}=\{y_{1},\ldots,y_{n}\}$ denote the vector with all data points (evidence) available and $\boldsymbol{\vartheta}$ denote the vector of the basic parameters $\rho_{gr}$, $\pi_{gr}$, $\delta_{gr}$. If $L_{i}\boldsymbol{(}\boldsymbol{\vartheta}\boldsymbol{;}y_{i})$ denotes the likelihood contribution from $y_{i}$ to (elements of) the basic parameter vector $\boldsymbol{\vartheta}$, then we derive the following likelihood model:

$$L(\boldsymbol{\vartheta};\boldsymbol{y})=\prod_{i=1}^{n} L_{i}(\boldsymbol{\vartheta};y_{i})$$

Via maximum likelihood we can derive from this model estimates of the basic parameters. Under a Bayesian perspective, prior (sometimes imperfect or scarce) knowledge around the basic parameters, as expressed through some joint prior distribution $p(\boldsymbol{\vartheta})$, may be updated in the light of the observed data into a posterior distribution $p(\boldsymbol{\vartheta}|\boldsymbol{y})$ summarizing all information around the basic parameters $\boldsymbol{\vartheta}$: $p(\boldsymbol{\vartheta}|\boldsymbol{y})\propto p\left( \boldsymbol{\vartheta} \right)L(\boldsymbol{\vartheta};\boldsymbol{y})$. The prior-to-posterior updating mechanism that is employed here synthesizes all available data and results in posterior distributions that fully reflect both the sampling variability and the parameter uncertainty surrounding the model. The uncertainty in the estimation of $\rho_{gr}$, $\pi_{gr}$, $\delta_{gr}$ is then propagated through the posterior distributions of the number of people living with diagnosed or undiagnosed HIV infection. This is usually summarized via medians and 95% credible intervals (CrIs), providing a point estimate and a measure of its accuracy, respectively. The use of multiple data sources can lead to conflicting evidence; these were reconciled in MPES through feedback from epidemiologists and data suppliers. Additionally, a number of assumptions were introduced as detailed in [1].

**Supporting Information 1B: Data sources**

Supporting Information 1B contains region specific data and sources (denoted with superscripts 1, 2, … 26, 27) used in the MPES model.

**Table B.1: Available data for Amsterdam.**

| **Key population** | **Gender** | | | **Basic parameters** | | | | **Functional parameter** | | |  |
| --- | --- | --- | --- | --- | --- | --- | --- | --- | --- | --- | --- |
|  | |  | **Group size**  **(*ρ*)** | | **HIV prevalence**  **(*π*)** | **Proportion diagnosed (*δ*)** | | | | **Number diagnosed**^ç,18^ |  |
| MSM – STI clinic | |  | 0.15 of MSM^1^ | | 0.254^1^ | | 0.95^11^ | |  | | |
| MSM – total | |  | 0.10^2^ | | 0.097^8^ | |  | | 3596^**^ | | |
| IDU | | M | 0.0008-0.0026^3,4^ | | 0.021^3,9^ | | 1^11,17^ | | 4^***^ | | |
|  | | F | 0.00018-0.00060^3,4^ | | 0.043^3,9^ | | 1^11,17^ | | 1^***^ | | |
| FSW | |  | 0.019^5,6^ | | 0.015^10^ | | 0^10^ | |  | | |
| STI clinic – total | | M |  | | 0.0031^11^ | | 0^11^ | |  | | |
|  | | F |  | | 0^11^ | | 0^11^ | |  | | |
| STI clinic – SSA | | M | 0.0009^1^ | | 0.0035^1*^ | |  | |  | | |
|  | | F | 0.0009^1^ | | 0.0038^1*^ | |  | |  | | |
| STI clinic – CRB | | M | 0.0047^1^ | | 0.0043^1*^ | |  | |  | | |
|  | | F | 0.0045^1^ | | 0.0022^1*^ | |  | |  | | |
| STI clinic – rest | | M | 0.017^1^ | | 0.0013^1*^ | |  | |  | | |
|  | | F | 0.024^1^ | | 0.00065^1*^ | |  | |  | | |
| SSA (non-STI clinic) | | M | 0.034^7^ | | 0.0078^12^, 0.044^13^, 0.05^13^ | |  | | 181 | | |
|  | | F | 0.029^7^ | | 0^12^, 0.044^13^, 0.05^13^ | |  | | 250 | | |
| CRB (non-STI clinic) | | M | 0.092^7^ | | 0.0047^12^, 0.006^13,14^, 0.012^13,14^ | |  | | 116 | | |
|  | | F | 0.106^7^ | | 0.0040^12^, 0.006^13,14^, 0.012^13,14^ | |  | | 98 | | |
| Pregnant women – non-migrant | |  |  | | 0.0006^7,15,16^ | | 0.83^15,16^ | |  | | |
| Pregnant women – migrant | |  |  | | 0.0093^7,15,16^ | | 0.80^15,16^ | |  | | |

Grey indicates the same data as used in the 2007 HIV prevalence estimate (Van Veen *et al*. 2011). MSM denotes men who have sex with men, IDU injecting drug users, FSW female sex workers, SSA people from sub-Saharan Africa, CRB people from former Netherlands Antilles and Surinam, M males and F females.

Total numbers of Amsterdam residents are 304,147 (M) and 308,855 (F).

^ç^ Total number diagnosed for each gender is 4.262 (M) and 659 (F), also including 89 (M) and 19 (F) cases of unknown exposure and 276 (M) and 291 (F) cases being a mixture of respectively male and female registered infections for IDU, FSW (F only), STI clinic – rest and key population at low risk.

^*^ Data inform minimum prevalences, due to STI clinic attendees opt-out fractions: 0.018 (MSM), 0.014 (M SSA), 0.011 (F SSA), 0.011 (M CRB), 0.016 (F CRB), 0.070 (M rest) and 0.175 (F rest),

^**^ Registered cases (MSM) are underestimated by a fraction of 492/518 = 0.95. This value is based on the Schorer Monitor 2011 where 95% of all HIV-positive MSM reported to be in care and is used for all three regions^19^. The respondents are a combination of people tested less than a year ago (15%) and more than one year ago (85%).

^***^ For IDU, the number diagnosed only includes new diagnoses between 2008 and 2012. All IDUs diagnosed and in care before 2008 are attributed to the mixed category (see ^ç^). Please note that regional numbers of new HIV diagnoses in IDU are based on the location of the treatment centre, rather than areas of residence as is used for the other key populations at higher risk. **Table B.2: Available data for Rotterdam.**

| **Key population** | **Gender** | | | **Basic parameters** | | | | **Functional parameter** | | |  |
| --- | --- | --- | --- | --- | --- | --- | --- | --- | --- | --- | --- |
|  | |  | **Group size**  **(*ρ*)** | | **HIV prevalence**  **(*π*)** | **Proportion diagnosed (*δ*)** | | | | **Number diagnosed**^ç,18^ |  |
| MSM – STI clinic | |  | 0.06 - 0.22 of MSM^1^ | | 0.095^1^ | |  | |  | | |
| MSM – total | |  | 0.035^2^ - 0.122^20^ | |  | |  | | 748^**^ | | |
| IDU | | M | 0.00095^4,17^ | | 0.020^17^ | | 0.31^24^ | | 4^***^ | | |
|  | | F | 0.00012^4,17^ | | 0.020^17^ | | 0.69^24^ | | 1^***^ | | |
| FSW | |  | 0.018^21^ | | 0.025^22~^ | | 0^10^ | |  | | |
| STI clinic – total | | M |  | | 0.0066^23^ | | 1^23^ | |  | | |
|  | | F |  | | 0.0054^23^ | | 1^23^ | |  | | |
| STI clinic – SSA | | M | 0.0013^1^ | | 0^1*^ | |  | |  | | |
|  | | F | 0.0016^1^ | | 0.0060^1*^ | |  | |  | | |
| STI clinic – CRB | | M | 0.0039^1^ | | 0.0083^1*^ | |  | |  | | |
|  | | F | 0.0045^1^ | | 0.0032^1*^ | |  | |  | | |
| STI clinic – rest | | M | 0.010^1^ | | 0.0020^1*^ | |  | |  | | |
|  | | F | 0.013^1^ | | 0.0030^1*^ | |  | |  | | |
| SSA (non-STI clinic) | | M | 0.046^7^ | | 0.0014^12^, 0.044^13^, 0.05^13^ | |  | | 87 | | |
|  | | F | 0.045^7^ | | 0^12^, 0.044^13^, 0.05^13^ | |  | | 131 | | |
| CRB (non-STI clinic) | | M | 0.117^7^ | | 0.033^12^, 0.006^13,14^, 0.012^13,14^ | | 0^12^ | | 72 | | |
|  | | F | 0.135^7^ | | 0.0089^12^, 0.006^13,14^, 0.012^13,14^ | | 0^12^ | | 67 | | |
| Pregnant women – non-migrant | |  |  | | 0.0003^7,15,16^ | | 0.5^15,16^ | |  | | |
| Pregnant women – migrant | |  |  | | 0.0041^7,15,16^ | | 0.71^15,16^ | |  | | |

Grey indicates the same data as used in the 2007 HIV prevalence estimate (Van Veen *et al*. 2011). MSM denotes men who have sex with men, IDU injecting drug users, FSW female sex workers, SSA people from sub-Saharan Africa, CRB people from former Netherlands Antilles and Surinam, M males and F females.

Total numbers of Rotterdam residents are 225,368 (M) and 225,548 (F).

^ç^ Total number diagnosed for each gender is 1052 (M) and 320 (F), also including 27 (M) and 15 (F) cases of unknown exposure and 114 (M) and 106 (F) cases being a mixture of respectively male and female registered infections for IDU, FSW (F only), STI clinic – rest and key population at low risk.

^*^ Data inform minimum prevalences, due to STI clinic attendees opt-out fractions: 0.039 (MSM), 0.043 (M SSA), 0.062 (F SSA), 0.028 (M CRB), 0.070 (F CRB), 0.081 (M rest) and 0.166 (F rest).

^**^ Registered cases (MSM) are underestimated by a fraction of 492/518 = 0.95. This value is based on the Schorer Monitor 2011 where 95% of all HIV-positive MSM reported to be in care and is used for all three regions^19^. The respondents are a combination of people tested less than a year ago (15%) and more than one year ago (85%).

^***^ For IDU, the number diagnosed only includes new diagnoses between 2008 and 2012. All IDUs diagnosed and in care before 2008 are attributed to the mixed category (see ^ç^). Please note that regional numbers of new HIV diagnoses in IDU are based on the location of the treatment centre, rather than areas of residence as is used for the other key populations at higher risk.

^~^ Same source as Van Veen *et al*. 2011, but SSA are included here due to the hierarchical structure of the model.

**Table B.3: Available data for the rest of the Netherlands (excluding Amsterdam and Rotterdam).**

| **Key population** | **Gender** | | | **Basic parameters** | | | | **Functional parameters** | | |
| --- | --- | --- | --- | --- | --- | --- | --- | --- | --- | --- |
|  | |  | **Group size**  **(*ρ*)** | | **HIV prevalence**  **(*π*)** | **Proportion diagnosed (*δ*)** | | | **Diagnosed HIV prevalence (*πδ*)** | **Number diagnosed**^ç,18^ |
| MSM – STI clinic | |  | 0.09 - 0.11 of MSM^1^ | | 0.118^1^ | |  | |  |  |
| MSM – total | |  | 0.025^25^ - 0.030^26^ | |  | |  | | 0.077^25^ -0.151^19^ | 5514^**^ |
| IDU | | M | 0.0002^4^ | | 0.050^24^ | | 0.435^24^ | |  | 18^***^ |
|  | | F | 0.0000^4^ | | 0.035^24^ | | 0.625^24^ | |  | 2^***^ |
| FSW | |  | 0.0015^27^ – 0.0031^27^ | | 0.0000^22^ | |  | |  |  |
| STI clinic – SSA | | M | 0.0001^1^ | | 0.0059^1^ | |  | |  |  |
|  | | F | 0.0001^1^ | | 0.0152^1^ | |  | |  |  |
| STI clinic – CRB | | M | 0.0004^1^ | | 0.0026^1^ | |  | |  |  |
|  | | F | 0.0004^1^ | | 0.0037^1^ | |  | |  |  |
| STI clinic – rest | | M | 0.0043^1^ | | 0.0030^1^ | |  | |  |  |
|  | | F | 0.0063^1^ | | 0.0016^1^ | |  | |  |  |
| SSA (non-STI clinic) | | M | 0.0093^7^ | | 0.0067^12^, 0.044^13^, 0.05^13^ | | 0^12^ | |  | 531^¥^ |
|  | | F | 0.0085^7^ | | 0.0172^12^, 0.044^13^, 0.05^13^ | | 0^12^ | |  | 951^¥^ |
| CRB (non-STI clinic) | | M | 0.0215^7^ | | 0.027^12^ , 0.006^13,14^, 0.012^13,14^ | | 0^12^ | |  | 137^¥^ |
|  | | F | 0.0237^7^ | | 0.0130^12^, 0.006^13,14^, 0.012^13,14^ | | 0^12^ | |  | 180^¥^ |
| Pregnant women – non-migrant | |  |  | | 0.0001^7,15,16^ | | 0.882^15,16^ | |  |  |
| Pregnant women – migrant | |  |  | | 0.0057^7,15,16^ | | 0.881^15,16^ | |  |  |
| Remaining low risk | | M | 1 - key populations | |  | |  | | 0.0009^25^ |  |
|  | | F | 1 - key populations | |  | |  | | 0.0005^25^ |  |

Grey indicates the same data as used in the 2007 HIV prevalence estimate (Van Veen *et al*. 2011). MSM denotes men who have sex with men, IDU injecting drug users, FSW female sex workers, SSA people from sub-Saharan Africa, CRB people from former Netherlands Antilles and Surinam, M males and F females.

Total numbers of residents in the rest of the Netherlands excluding Amsterdam and Rotterdam are 5,508,360 (M) and 5,446,844 (F).

^ç^ Total number diagnosed for each gender is 7.624 (M) and 2.172 (F), also including 268 (M) and 71 (F) cases of unknown exposure and 1156 (M) and 968 (F) cases being a mixture of respectively male and female registered infections for IDU, FSW (F only), STI clinic – rest and key population at low risk.

^*^ Data inform minimum prevalences, due to STI clinic attendees opt-out fractions: 0.014 (STI MSM), 0.043 (M SSA), 0.053 (F SSA), 0.026 (M CRB), 0.053 (F CRB), 0.145 (M rest) and 0.266 (F rest)

^**^ Registered cases (MSM) are underestimated by a fraction of 492/518 = 0.95. This value is based on the Schorer Monitor 2011 where 95% of all HIV-positive MSM reported to be in care and is used for all three regions^19^. The respondents are a combination of people tested less than a year ago (15%) and more than one year ago (85%).

^***^ For IDU, the number diagnosed only includes new diagnoses between 2008 and 2012. All IDUs diagnosed and in care before 2008 are attributed to the mixed category (see ^ç^). Please note that regional numbers of new HIV diagnoses in IDU are based on the location of the treatment centre, rather than areas of residence as is used for the other key populations at higher risk..

^¥^ Information on place of HIV diagnosis of patients in care in 2012 was used to distribute migrants in care by STI clinic attendance status: 0.20 (SSA M), 0.23 (SSA F), 0.17 (CRB M) and 1.0 (CRB F) were diagnosed in STI clinics.

**Table B.4: Data sources for parameters in Tables B.1-B.3**

| 1 | National registry of all new STI consultations in STI clinics (SOAP) in 2012, separate sub-analyses for respondents from Amsterdam, Rotterdam and the rest of the Netherlands |
| --- | --- |
| 2 | For Amsterdam: data from the Amsterdam Health Monitor, a random sample of the Amsterdam population in 2012. For Rotterdam: data from the Rotterdam Health Monitor, a random sample of the Rotterdam population in 2008 |
| 3 | Trimbos Institute, National Drug monitor 2012 |
| 4 | Cruts G, van Laar M, Buster M. “Aantal en kenmerken van problematische opiatengebruikers in Nederland” [in Dutch]. Utrecht, the Netherlands: Trimbos instituut/GGD Amsterdam; 2013 |
| 5 | Van Wijk A, Nieuwenhuis A, van Tuyn D, van Ham T, Kuppens J, Ferwerda H. “Kwetsbaar beroep: een onderzoek naar de prostitutiebranche in Amsterdam” [in Dutch]. Bureau Beke; 2010 |
| 6 | Municipality of Amsterdam, report on prostitution policy in Amsterdam 2000 |
| 7 | Data from National Office of Statistics (CBS) ([www.statline.nl](http://www.statline.nl)). Registered inhabitants from Amsterdam, Rotterdam and the rest of the Netherlands in 2012, aged 15-70 years, according to their country of origin |
| 8 | Data from Amsterdam Cohort Study in MSM since 1984, data used for MSM who were in follow-up in 2012 ([www.amsterdamcohortstudies.org](http://www.amsterdamcohortstudies.org)) |
| 9 | Schreuder I, van der Sande MAB, de Wit M, Bongaerts M, Boucher CAB, Croes EA, van Veen MG. Seroprevalence of blood-borne viral infections among opioid drug users on methadone treatment in the Netherlands. Harm Reduct J 2010,7:25 |
| 10 | van Veen MG, Götz HM. van Leeuwen PA, Prins M. van de Laar MJW. HIV and sexual risk behaviour among commercial sex workers in the Netherlands. Archives of sexual behavior 2010,39:714-723 |
| 11 | Anonymous unlinked HIV testing among STI clinic attendees in STI clinic in Amsterdam in 2012 (DWAR), STI clinic Amsterdam, the Netherlands |
| 12 | Data from anonymous unlinked HIV survey among migrants (from Sub-Sahara Africa and the Caribbean), recruited at (multicultural) social venues. Migrants living in Amsterdam were recruited in 2003-2004, in Rotterdam in 2006, and in the Hague (used as proxy for the rest of the Netherlands) in 2005 |
| 13 | Joint United Nations Programme on HIV/AIDS (UNAIDS). Global Report: UNAIDS report on the global AIDS epidemic 2013. Geneva, Switzerland: UNAIDS; 2013 |
| 14 | De Wolf F. HIV Treatment and Resistance in Curaçao. Presented at the 8th HIV Monitoring Update; May 2-3, 2012; Curaçao |
| 15 | Data from screening of pregnant women in 2010-2011: Van der Ploeg CBP, van der Pal SM, van Gameren-Oosterom HBM, Oomen P. “Procesmonitoring prenatale screening infectieziekten en Erytrocytenimmunisatie 2009-2011” [In Dutch]. Leiden: TNO; 2012 |
| 16 | Data of HIV infected women identified through screening of pregnant women notified to the HIV Monitoring Foundation (SHM) between 2008 and 2012 |
| 17 | Van Veen MG & Schreuder I. “Infectieziektebestrijding in de verslavingszorg in Nederland” [In Dutch] Bilthoven: RIVM; 2009 |
| 18 | Data of all registered HIV cases in care, alive, living in the Netherlands and in follow-up at treatment centres in 2012, subdivided into regions based on living area of the cases; data from the ATHENA cohort from the HIV Monitoring Foundation (SHM). |
| 19 | van Empelen P, van Berkel M, Roos E, Zuilhof W. Schorer Monitor 2011. Amsterdam, the Netherlands: Schorer Foundation; 2012 http://old.mantotman.nl/mannen_met_hiv_606.html |
| 20 | Rutgers WPF. Sexual health of the Netherlands, a national sample with sub-analyses of respondents from Rotterdam in 2006. RNG, the Netherlands |
| 21 | Verwey Jonker Institute 2007. Report on prostitution policy in Rotterdam in 2005/2006 [In Dutch] |
| 22 | Data from anonymous unlinked HIV survey among commercial sex workers in the Netherlands. For Rotterdam, sex workers were recruited in brothels, clubs and street-based prostitution zone in 2002-2003. For the rest of the Netherlands, data were available only for sex workers recruited in brothels, clubs and street-based prostitution zone in The Hague (used as proxy for the rest of the Netherlands) in 2005 |
| 23 | Anonymous unlinked HIV testing data among STI clinic attendees in STI clinic in Rotterdam in 2005-2007 (ROTan), STI clinic Rotterdam, the Netherlands |
| 24 | Data from anonymous unlinked HIV survey among IDU in different cities in the Netherlands, recruited in- and out-side treatment facilities for IDU. In Rotterdam in 2002-2003, and in seven other cities (data used for the rest of the Netherlands) in the period 1996-2000 |
| 25 | PIENTER project. Prevalence of HIV viral markers in the Dutch population in 2006/2007: a population-based sero-surveillance study population, analyses separately for respondents from the rest of the Netherlands |
| 26 | Keuzenkamp S, Kooiman N, van Lisdonk J. “Niet te ver uit de kast – Ervaringen van home- en biseksuelen in Nederland” [In Dutch]. The Hague, the Netherlands: the Netherlands institute for social research; 2012 |
| 27 | van der Helm T, van Mens L. Mobility in prostitution in The Netherlands 1998-1999. Technical report, Municipal Health Service Amsterdam, 1999. An Inventory Done Under the Auspices of EUROPAP-TAMPEP 1998-1999 |

**Supporting Information 1C: PLWHA and number undiagnosed**

For completeness, we report here the number of PLWHA and the number of people with an undiagnosed HIV infection per region and the two largest groups: MSM and migrants.

**Table C.1: Estimates of the number of PLWHA and the number of people with an undiagnosed HIV infection for MSM and migrants by region in the Netherlands for 2007 and 2012.**

|  | **People living with HIV/AIDS (95% CrI)** | | **People living with undiagnosed HIV/AIDS (95% CrI)** | |
| --- | --- | --- | --- | --- |
|  | **2007** | **2012** | **2007** | **2012** |
| **Total** | **21,444 (17,204 – 28,694)** | **24,350 (20,420 – 31,280)** | **8,520 (4,304 – 15,751)** | **8,318 (4,406 – 15,260)** |
| Amsterdam | 5,120 (4,720 **–** 5,777) | 5,649 (5,290 – 6,238) | 885 (512 – 1,521) | 769 (439 – 1,356) |
| Rotterdam | 2,444 (2,027 **–** 3,112) | 2,351 (2,040 – 2,859) | 1,118 (705 – 1,783) | 1,005 (706 – 1,514) |
| Rest of NL | 13,807 (9,723 **–** 20,990) | 16,290 (12,400 – 23,080) | 6,421 (2,360 – 13,603) | 6,493 (2,620 – 13,310) |
| **MSM** | **11,758 (8,670 – 17,573)** | **15,590 (12,070 – 21,780)** | **4,028 (969 – 9,848)** | **4,856 (1,359 – 11,050)** |
| Amsterdam | 3,404 (3,138 **–** 3,931) | 4,263 (3,978 – 4,769) | 351 (140 – 854) | 368 (132 – 869) |
| Rotterdam | 839 (679 – 1,092) | 1,169 (1,020 – 1,339) | 148 (24 – 413) | 364 (232 – 533) |
| Rest of NL | 7,461 (4,452 – 13,220) | 10,140 (6,641 – 16,250) | 3,470 (463 – 9,213) | 4,094 (614 – 10,190) |
| **SSA** | **4,299 (3,408 – 5,519)** | **3,761 (3,103 – 4,693)** | **2,146 (1,263 – 3,360)** | **1,795 (1,150 – 2,717)** |
| Amsterdam | 567 (458 – 748) | 512 (421 – 639) | 174 (90 – 350) | 166 (91 – 286) |
| Rotterdam | 712 (519 – 990) | 456 (347 – 603) | 454 (273 – 726) | 257(155 – 398) |
| Rest of NL | 2,990 (2,168 – 4,192) | 2,784 (2,144 – 3,692) | 1,484 (662 – 2,673) | 1,363 (733 – 2,268) |
| **CRB** | **1,283 (1,047 – 1,576)** | **1,083 (903 – 1,316)** | **576 (359 – 841)** | **476 (310 – 696)** |
| Amsterdam | 288 (226 – 383) | 225 (178 – 288) | 63 (27 – 148) | 55 (24 – 108) |
| Rotterdam | 377 (267 – 542) | 269 (200 – 371) | 221 (127 – 370) | 140 (80 – 233) |
| Rest of NL | 602 (433 – 850) | 580 (431 – 793) | 275 (112 – 502) | 272 (133 – 477) |

MSM: men having sex with men; SSA: Sub-Saharan Africans; CRB: Caribbeans; CrI: Credible interval; NL: Netherlands.

**Supporting Information 1D: Sensitivity analysis**

For some key populations at higher risk or data sources, new insights became available since 2007 that called for changes in the model structure and assumptions. Table D.1 provides an overview of all changes applied to the model and Table D.2 shows all corresponding input values used in the sensitivity analysis. The input values displayed under the “Main analysis” heading are the same values described throughout the main text and in Supporting Information 1. The input values informing the sensitivity analysis reflect more recent data (where available), while using the same definitions of key populations and model assumptions as adopted in 2007 [1].

Table D.3 shows the results of the sensitivity analyses. The prevalence was not sensitive to the model alterations. In all scenarios, the prevalence remained around 0.2% (95% CrI 0.2% – 0.3%). The number of PLWHA increased from 24,350 (95% CrI: 20,420 – 31,280) in the main analyses to 25,150 (95% CrI: 21,670 – 32,690) in the sensitivity analysis adopting all 2007 model assumptions. This increase was mainly due to the inclusion of all people registered in care (including those that were lost to follow-up) and to the change in definition of IDU. The proportion undiagnosed was 30.4% (95%CrI: 19.3% – 46.5%) when including all 2007 model assumptions. This is somewhat lower than the 34.2% (95% CrI: 21.6% – 48.8%) obtained in the main analyses. This lower proportion diagnosed can be largely explained by the definition change of IDU.

With a Bayesian modelling framework, the posterior mean deviances can be used as a measure of goodness of fit [2]. Generally, the higher the deviances, the stronger is the potential lack of fit. The posterior mean deviances regarding the proportion undiagnosed and prevalence among migrant populations, and pregnant migrants were generally higher adopting all 2007 model assumptions compared to the 2012 assumptions. Most other deviances were close to one in both estimates. The largest decline in deviance was observed in pregnant migrant women in the rest of the Netherlands, the deviance for the prevalence decreased from 93.3 using the 2007 model assumptions to 6.3 using the 2012 assumptions. Less remarkable differences were observed among migrant populations, with, for example, deviances regarding the proportion diagnosed decreasing from 5.4 to 3.9 for SSA visiting the STI-clinic in Rotterdam.

**Table D.1: Overview of all changes to the model**

| Key population | Year | |
| --- | --- | --- |
|  | 2007 | 2012 |
| IDU | Definition: ever injected drugs | Definition: current IDU (< 6 months since last injecting). |
| All | Number of cases in care including those lost to follow-up | Number of cases in care excluding those lost to follow-up |
| Low risk | Including blood donors | Excluding blood donors |
| STI clinic – rest | Individuals opting out of HIV testing are at higher risk compared to those opting –in. | Individuals opting out of HIV testing are at zero risk compared to those opting –in. |
| Pregnant women | No data on diagnosed infections Rotterdam and Rest NL | Data on diagnosed infections Rotterdam and Rest NL |
|  | The denominator in the HIV prevalence estimate for non-migrant women Rest NL represent all other nationalities than Dutch | The denominator in the HIV prevalence estimate for non-migrant women Rest NL represent SSA and CRB nationalities only |

IDU denotes injecting drug users, SSA people from sub-Saharan Africa, CRB people from former Netherlands Antilles and Surinam and NL the Netherlands.

**Table D.2: Overview of all input values used in the sensitivity analysis**

| Key population | Parameter | | | | Sub-  group | Input values | | | | | |
| --- | --- | --- | --- | --- | --- | --- | --- | --- | --- | --- | --- |
|  |  | | | |  | Main analysis^*^ | | | Sensitivity analysis^**^ | | |
| *Definition IDU* | | | | | | | | | | | |
|  |  | | | |  | Amsterdam | Rotterdam | Rest NL | Amsterdam | Rotterdam | Rest NL |
| IDU | *ρ* | | | | M | 245– 783 | 214 | 1135 | 706 – 862 | 534 | 3671 |
|  |  | | | | F | 57 – 184 | 26 | 249 | 165 – 202 | 66 | 806 |
|  | *π* | | | | M | 0.02 | 0.02 | 0.05 | 0.27 | 0.09 | 0.05 |
|  |  | | | | F | 0.04 | 0.02 | 0.04 | 0.20 | 0.14 | 0.04 |
|  | *δ* | | | | M | 1 | 0.31 | 0.43 | 0.69 | 0.31 | 0.43 |
|  |  | | | | F | 1 | 0.69 | 0.63 | 0.5 | 0.69 | 0.63 |
|  | *ρδ* | | | | M | - |  |  | 0.14^a^ |  |  |
|  |  | | | | F | - |  |  | 0.14^a^ |  |  |
| *Cases in care* | | | | | | | | | | | |
|  |  | | | |  | Amsterdam | Rotterdam | Rest NL | Amsterdam | Rotterdam | Rest NL |
| MSM | *μ* | | | | M | 3596 | 748 | 5514 | 3784 | 786 | 5707 |
| IDU |  | | | | M | 4 | 4 | 18 | 4 | 4 | 18 |
|  |  | | | | F | 1 | 1 | 2 | 1 | 1 | 2 |
| SSA |  | | | | M | 181 | 87 | 531 | 222 | 124 | 697 |
|  |  | | | | F | 250 | 131 | 951 | 297 | 160 | 1156 |
| CRB |  | | | | M | 116 | 72 | 137 | 131 | 77 | 151 |
|  |  | | | | F | 98 | 67 | 180 | 107 | 73 | 189 |
| Unknown |  | | | | M | 89 | 27 | 268 | 122 | 33 | 304 |
|  |  | | | | F | 19 | 15 | 71 | 23 | 16 | 81 |
| Mixed ^ç^ |  | | | | M | 276 | 114 | 1156 | 300 | 129 | 1264 |
|  |  | | | | F | 291 | 106 | 968 | 312 | 115 | 1024 |
| *Blood donors* | | | | | | | | | | | |
|  |  | | | |  | Amsterdam | Rotterdam | Rest NL | Amsterdam | Rotterdam | Rest NL |
| Low risk | *π* | | | | M/F |  |  | - |  |  | 0.00^b^ |
| *Opting-out prevalence* | | | | | | | | | | | |
| STI clinic – rest | |  | | |  | Opting-out prevalence multiplication factor | | | | | |
|  | |  | | |  | Amsterdam | Rotterdam | Rest NL | Amsterdam | Rotterdam | Rest NL |
|  | |  | | |  | 0 | 0 | 0 | 0.5 | 0.5 | 0 |
| *Pregnant migrants* | | | | | | | | | | | |
|  | | |  |  | | Amsterdam | Rotterdam | Rest NL | Amsterdam | Rotterdam | Rest NL |
| Pregnant women | | | *δ* | Non-migrant | | 0.83 | 0.50 | 0.88 | 0.83 | - | - |
|  | | |  | Migrant | | 0.80 | 0.71 | 0.88 | 0.80 | - | - |
|  | | | *π* | Non-migrant | | 0.0006 | 0.0003 | 0.0001 | 0.0006 | 0.0003 | 0.0001^c^ |
|  | | |  | Migrant | | 0.0093 | 0.0041 | 0.0057 | 0.0093 | 0.0041 | 0.0024^c^ |

Grey indicates the same data as used in the 2007 HIV prevalence estimate (Van Veen *et al*. 2011). MSM denotes men who have sex with men, IDU injecting drug users, SSA people from sub-Saharan Africa, CRB people from former Netherlands Antilles and Surinam, M males and F females.

^ç^ Cases being a mixture of respectively male and female registered infections for IDU, Female sex workers (F only), STI clinic – rest and key population at low risk

^*^ Data values and sources are the same as displayed in Tables B.1-B.3.

^**^ Data sources are the same as displayed in Tables B.1-B.3. Additional sources include:

^a^ Amsterdam Cohort studies on drug users (since 1985); data used from drug users who ever injected drugs and were in follow-up in 2009.

^b^ Data from blood donor screening on HIV (Sanquin 2012, Netherlands).

^c^ Subdivision of pregnant women in migrant and non-migrant groups was based on data from National Office of Statistics (CBS) on ethnicity of mothers giving living births in 2012 in the Netherlands (Dutch vs non-Dutch).

**Table D.3: Model outcomes when reverting model adaptations back to the 2007 model set-up**

| **Model adaptation** | **Total prevalence, % (95% CrI)** | **Total number of PLWHA (95% CrI)** | **Total proportion undiagnosed,**  **% (95% CrI)** |
| --- | --- | --- | --- |
| Estimates from main analyses | 0.2 (0.2 – 0.3) | 24,350 (20,420 – 31,280) | 34.2 (21.6 – 48.8) |
| *All adaptations:* |  |  |  |
| All at the same time | 0.2 (0.2 – 0.3) | 25,150 (21,670 – 32,690) | 30.4 (19.3 – 46.5) |
| *Single adaptation:* ^ç^ |  |  |  |
| Change of IDU definition to current users | 0.2 (0.2 – 0.3) | 25,800 (20,510 – 38,930) | 30.9 (19.0 – 46.3) |
| Exclusion of people lost to follow-up in care | 0.2 (0.2 – 0.3) | 25,960 (21,890 – 33,440) | 33.4 (21.1 – 48.2) |
| Exclusion of blood donors | 0.2 (0.2 – 0.3) | 24,710 (20,170 – 31,410) | 35.2 (20.6 – 49.0) |
| Opting-out prevalence STI clinic – rest group set to zero | 0.2 (0.2 – 0.3) | 24,640 (20,430 – 31,770) | 35.0 (21.7 – 49.6) |
| Pregnant women: including data on diagnosed infections for Rotterdam and Rest NL and data on HIV prevalence for Rest NL migrants represent SSA and CRB (oppose to all non-Dutch nationalities) | 0.2 (0.2 – 0.3) | 23,670 (19,890 – 30,260) | 32.6 (19.8 – 47.2) |

IDU denotes injecting drug users, SSA people from sub-Saharan Africa, CRB people from former Netherlands Antilles and Surinam and NL the Netherlands.

^ç^ In this analysis, only one model adaptation at the time is reversed to the 2007 model set-up (Table D.1), all remaining adaptations are not reversed and the 2012 set-up is used.

**References**

1. van Veen MG, Presanis AM, Conti S, et al*.* National estimate of HIV prevalence in the Netherlands: comparison and applicability of different estimation tools. AIDS. 2011;25:229-237.
2. Spiegelhalter DJ, Best NG, Carlin BL, et al. Bayesian measures of model complexity and fit. JR Stat Soc Ser B Stat Methodol. 2002;64:583-639.
